# Neuroprotective Effects of Sulforaphane on Cholinergic Neurons in Mice with Alzheimer’s Disease-Like Lesions

**DOI:** 10.3390/ijms150814396

**Published:** 2014-08-18

**Authors:** Rui Zhang, Jingzhu Zhang, Lingduo Fang, Xi Li, Yue Zhao, Wanying Shi, Li An

**Affiliations:** 1 Department of Nutrition and Food Hygiene, School of Public Health, China Medical University, Shenyang 110001, China; E-Mails: z_zhangrui@163.com (R.Z.); zhangjz0107@163.com (J.Z.); fanglingduo@163.com (L.F.); rabbitto89@163.com (X.L.); yuezhao2000@163.com (Y.Z.); 2 Department of Clinical Nutrition, First Affiliated Hospital, China Medical University, Heping District, Shenyang 110001, China; E-Mail: shiwanying2019@gmail.com

**Keywords:** Alzheimer’s disease, sulforaphane, neurobehavior, cholinergic neuron

## Abstract

Alzheimer’s disease (AD) is a common neurodegenerative disease in elderly individuals, and effective therapies are unavailable. This study was designed to investigate the neuroprotective effects of sulforaphane (an activator of NF-E2-related factor 2) on mice with AD-like lesions induced by combined administration of aluminum and d-galactose. Step-down-type passive avoidance tests showed sulforaphane ameliorated cognitive impairment in AD-like mice. Immunohistochemistry results indicated sulforaphane attenuated cholinergic neuron loss in the medial septal and hippocampal CA1 regions in AD-like mice. However, spectrophotometry revealed no significant difference in acetylcholine level or the activity of choline acetyltransferase or acetylcholinesterase in the cerebral cortex among groups of control and AD-like mice with and without sulforaphane treatment. Sulforaphane significantly increased the numbers of 5-bromo-2'-deoxyuridine-positive neurons in the subventricular and subgranular zones in AD-like mice which were significantly augmented compared with controls. Atomic absorption spectrometry revealed significantly lower aluminum levels in the brains of sulforaphane-treated AD-like mice than in those that did not receive sulforaphane treatment. In conclusion, sulforaphane ameliorates neurobehavioral deficits by reducing cholinergic neuron loss in the brains of AD-like mice, and the mechanism may be associated with neurogenesis and aluminum load reduction. These findings suggest that phytochemical sulforaphane has potential application in AD therapeutics.

## 1. Introduction

Alzheimer’s disease (AD) is an age-related neurodegenerative disorder leading to the most common form of dementia [1]. In 2000, 25 million people were diagnosed with AD worldwide, a number that is expected to increase to 114 million by 2050, suggesting a dramatic increase in the burden of AD among elderly individuals [2]. AD is a multifactorial disorder related to genetic and environmental factors [3]. Its pathological hallmarks are extracellular plaques and intracellular neurofibrillary tangles, which consist of β-amyloid (Aβ) and hyperphosphorylated *tau*, respectively [4,5].

The onset of AD is insidious, and the earliest notable symptom is mild memory impairment. This symptom gradually progresses to severe dementia, which affects multiple cognitive and behavioral functions. Accumulating evidence shows that the progressive loss of memory and decline in cognitive function are due to the death of neurons, particularly cholinergic neurons, in brains affected by AD [6]. In addition, postmortem studies of the brains of patients with AD revealed low levels of the neurotransmitter acetylcholine (ACh) and the enzyme choline acetyltransferase (ChAT), which is responsible for ACh synthesis [7]. To slow down the progression of AD and improve cognitive function, researchers have attempted to restore the cholinergic balance through inhibition of cholinesterase-mediated ACh breakdown [6,8]. Several cholinesterase inhibitors have been approved for AD therapy, but these drugs have been shown to provide relatively modest benefits and to cause multiple side effects [8,9].

Due to their safety and efficacy, certain naturally occurring dietary phytochemicals have received considerable attention as alternative candidates for AD therapy [10]. Sulforaphane (SFN), an activator of NF-E2-related factor 2 (Nrf2), is a natural product isolated from cruciferous vegetables. Given its multi-biological activities, SFN may play beneficial roles in both delaying the initiation of diverse diseases and slowing down the progression of established diseases. Recently, Lee *et al.* [11] found that SFN protected against Aβ-induced oxidative cell death in SHSY5Y cells. Kim *et al.* [12] reported that SFN could ameliorate cognitive impairment and protect the brain from amyloidogenic damages in an Aβ-induced AD acute mouse model. However, the exact neuroprotective mechanism of SFN in AD, especially its effects on the cholinergic system, has not yet been documented. In this study, we thus investigated whether SFN could ameliorate cognitive deficits by protecting the brain’s cholinergic system from damage using an AD-like mouse model, in which AD-like lesions were induced by combined administration of aluminum and d-galactose [13].

## 2. Results and Discussion

### 2.1. Signs and Body Weight

**Figure 1:**
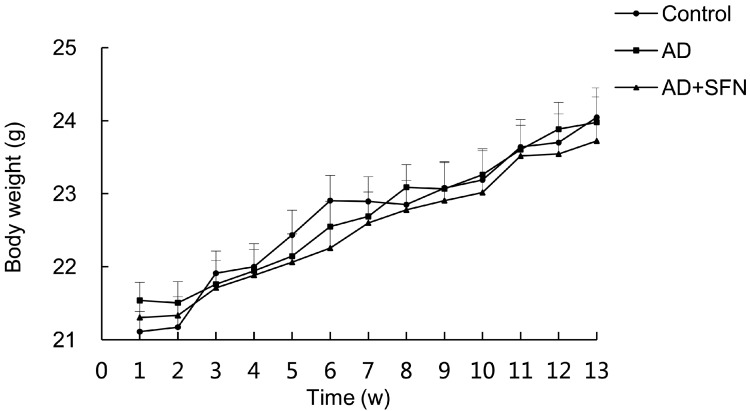
Measurement of mice body weight. During the treatment, no significant difference in bodyweight was observed among control, Alzheimer’s disease-like (AD-like)miceand sulforaphane-treated Alzheimer’s disease-like (AD+SFN-like) mice (*n* = 18; mean ± S.E.M.; One-way analysis of variance followed by *post* *hoc* least significant difference multiple comparison tests).

During the treatment, no significant sign of toxicity was observed in mice. No significant difference in body weight was observed among groups (Figure 1).

### 2.2. Analysis of Aluminum Level in the Mouse Brain

**Figure 2:**
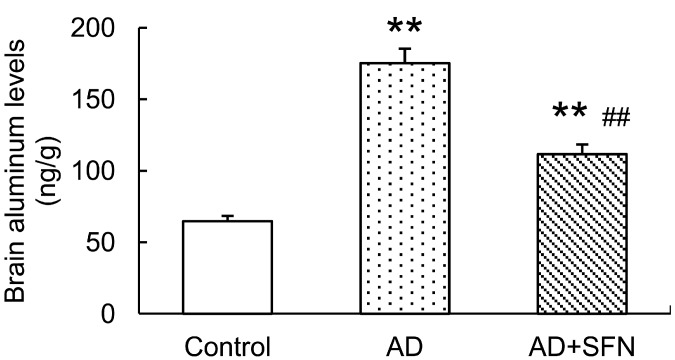
Analysis of aluminum level in the mouse brain. Brain aluminum levels were significantly higher inAD-like mice and AD+SFN-like mice than in controls; AD-like mice with sulforaphanetreatment exhibited lower brain aluminum levelsthanAD-like mice without sulforaphane treatment. (*n* = 10; means ± S.E.M.; One-way analysis of variance followed by *post hoc* least significant difference multiple comparison tests; ** *p* < 0.01 *versus* control, ## *p* < 0.01 *versus* AD).

Brain aluminum levels were significantly higher in AD-like mice with and without SFN treatment than in controls (*p* < 0.01). AD-like mice treated with SFN exhibited lower brain aluminum levels than AD-like mice without SFN treatment (*p* < 0.01; Figure 2).

### 2.3. Step-Down-Type Passive Avoidance Tests

**Figure 3:**
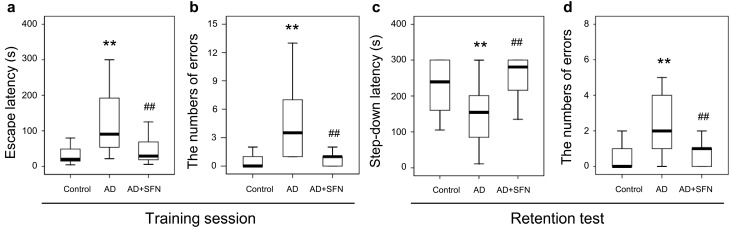
Step-down-type passive avoidance tests in control, AD-like mice and AD+SFN-like mice. In the training session, escape latency and the number of errors were significantly higher in AD-like mice than in controls; Sulforaphane markedly reduced escape latency and the number of errors in AD-like mice (**a**,**b**); In the retention test, shortened step-down latency and an increased number of errors were observed in AD-like mice compared with controls, while sulforaphane obviously increased step-down latency and reduced the number of errors in AD-like mice (**c**,**d**);No significant difference was detected between AD-like mice with sulforaphane treatment and controls in escape latency or the number of errors in the training session (**a**,**b**), or step-down latency and number of errors in the retention test (**c**,**d**). (*n* = 18; median andinterquartile range; Kruskal-Wallis non-parametric one way analysis of variance followed by followed by Mann-Whitney *U*-test (two-tailed); ** *p* < 0.01 *versus* control, ## *p* < 0.01 *versus* AD).

The results from step-down-type passive avoidance tests are shown in Figure 3. In the training session, escape latency and the number of errors were significantly higher in AD-like mice than in controls (*p* < 0.01); SFN markedly reduced escape latency and the number of errors in AD-like mice (*p* < 0.01). In the retention test, shortened step-down latency and an increased number of errors were observed in AD-like mice compared with controls (*p* < 0.05 and *p* < 0.01, respectively), while SFN obviously increased step-down latency and reduced the number of errors in AD-like mice (*p* < 0.01). No significant difference was detected between AD-like mice with SFN treatment and controls in escape latency or the number of errors in the training session, or step-down latency or the number of errors in the retention test.

### 2.4. Choline Acetyltransferase (ChAT) Immuno-Positive Neuron Assays

**Figure 4:**
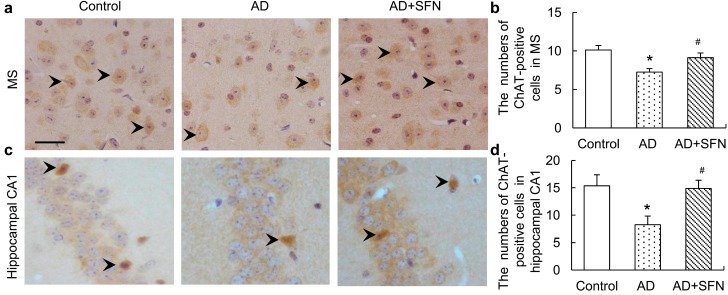
Immunohistochemistry assays of cholinergic neurons in themedial septal (MS) and hippocampal CA1 regions. Brown immunoreactive cells imply the cholinergic neurons in mice brain (see arrows, bars = 50 μm). The numbers of cholinergic neurons were markedly decreased in the MS (**a**) and hippocampal CA1 (**c**) regions of AD-like mice compared with controls and AD + SFN-like mice. However, the numbers of cholinergic neurons in these regions did not differ significantly between groups of control and AD-like mice with sulforaphane treatment; (**b**) and (**d**) showed quantitative assessments of choline acetyltransferase (ChAT) immuno-positive neurons in MS and hippocampus CA1, respectively. (*n* = 8; means ± S.E.M.; One-way analysis of variance followed by *post hoc* least significant difference multiple comparison tests; * *p* < 0.05 *versus* control, # *p* < 0.05 *versus* AD; magnify 400×; all cholinergic neurons in hippocampus CA1 were counted; number of cholinergic neurons of each field of vision in the MS was counted).

ChAT immunohistochemistry results are shown in Figure 4. Brown immunoreactive cells indicated the presence of imply cholinergic neurons in mouse brains. The numbers of cholinergic neurons were markedly decreased in the medial septal (MS) and hippocampal CA1 regions of AD-like mice compared with controls and AD-like mice treated with SFN (*p* < 0.05). However, the numbers of cholinergic neurons in these regions did not differ significantly between control and AD-like mice with SFN treatment.

### 2.5. 5-Bromo-2'-deoxyuridine Immuno-Positive Cell Assays

**Figure 5:**
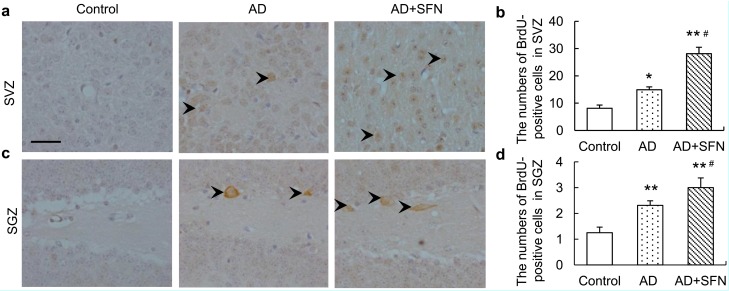
Immunohistochemistry assays of5-bromo-2'-deoxyuridine (BrdU)-positive neurons in thesubventricular zone (SVZ) and subgranular zone (SGZ).Brown immunoreactive cells imply the BrdU-positive neurons in mice brain (see arrows, bars = 100 μm). Compared with controls, significantlymore BrdU-positive cells were observed in the SVZ (**a**) and SGZ (**c**) of AD-like mice and AD+SFN-like mice. Moreover, sulforaphane significantly increased the numbers of BrdU-positive cells in the SVZ (**a**) and SGZ (**c**) of AD-like mice;(**b**) and (**d**) showed quantitative assessments of BrdU-positive cells in SVZ and SGZ, respectively. (*n* = 8; means ± S.E.M.; One-way analysis of variance followed by *post hoc* least significant difference multiple comparison tests; * *p* < 0.05 *versus* control, # *p* < 0.05 *versus* AD; ** *p* < 0.05 *versus* control; magnify 200×; numbers of BrdU-positive neurons of each field of vision in both SVZ and SGZ were counted).

5-Bromo-2'-deoxyuridine (BrdU), a proliferation marker, naturally incorporates into proliferating cells as a thymidine analog. BrdU immunohistochemistry results are shown in Figure 5. Compared with controls, significantly more BrdU-positive cells were observed in the subventricular zone (SVZ) and subgranular zone (SGZ) of AD-like mice with and without SFN treatment (*p* < 0.05 and *p* < 0.01, respectively). Moreover, SFN significantly increased the numbers of BrdU-positive cells in the SVZ and SGZ of AD-like mice (*p* < 0.05).

### 2.6. Acetylcholine (Ach) Level and Activities of ChAT and Acetylcholinesterase in the Cerebral Cortex

**Figure 6:**
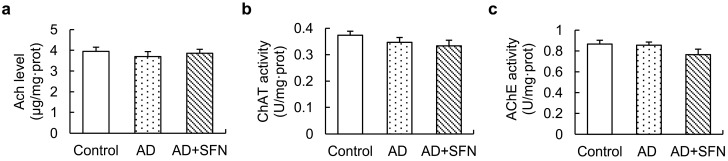
Acetylcholine (Ach)level and activities of ChAT and acetylcholinesterase (AChE) in the cerebral cortex.ACh level (**a**) and activities of ChAT (**b**) and AChE (**c**) in the cerebral cortex did not differ significantly among controls, AD-like mice and AD + SFN-like mice. (*n* = 10; means ± S.E.M.; One-way analysis of variance followed by *post hoc* least significant differencemultiple comparison tests).

Although ACh level showed a decreasing trend in AD-like mice, no significant alteration was found, and activities of ChAT and acetylcholinesterase (AChE) in the cerebral cortex did not differ significantly among groups (Figure 6).

### 2.7. Discussion

Animal models have been commonly used in defining critical disease-related mechanisms and for the preclinical evaluation of potential therapeutic interventions in AD. In this study, mice with AD-like lesions induced by the combined administration of aluminum and d-galactose [13] were used to investigate the anti-AD effects of SFN. SFN ameliorated cognitive impairment and attenuated cholinergic neuron loss in the MS and hippocampal CA1 regions in AD-like mice. However, no significant difference in ACh level or the activity of ChAT or AChE in the cerebral cortex was detected among groups of control and AD-like mice with and without SFN treatment. Moreover, SFN markedly increased the numbers of BrdU-positive neurons in the SVZ and SGZ of AD-like mice, which were significantly augmented compared with controls. Additionally, brain aluminum levels were significantly lower in AD-like mice with than in those without SFN treatment.

Behavioral dysfunction, especially memory loss, is the prominent and early symptom in AD patients. The Morris water maze is commonly used to test hippocampal-dependent spatial learning and memory. Morris water maze results from a previous study and ours showed impaired spatial memory in AD-like mice subjected to combined d-galactose and aluminum treatment [13,14]. In this study, step-down-type passive avoidance tests conducted to investigate non-spatial long-term memory demonstrated reduced cognitive function in AD-like mice. Memory dysfunction is well known to be associated mainly with cholinergic neuron loss in several regions of the brain, especially the basal forebrain and hippocampal CA1 region [15,16,17]. Consistent with previous studies [18,19,20], ChAT (a specific marker for cholinergic cells) immunohistochemical results from this study indicated cholinergic neuron loss in the MS and hippocampal CA1 regions in AD-like mice.

The combined administration of aluminum (20 mg/kg, intragastrically, once per day) and d-galactose (120 mg/kg, injected subcutaneously, once per day) has been shown to decrease the whole-brain ACh level and activities of ChAT and AChE in mice after treatment for 10 weeks [13]. However, we detected no significant alteration in ACh level or the activity of ChAT or AChE in the cerebral cortex of AD-like mice. The reasons for these inconsistent findings remain unclear. Generally, the literature reflects disagreement on ACh level and ChAT and AChE activities in the AD brain. Reduced ACh level and ChAT activity and increased AChE activity have been reported in the brains of patients with severe AD compared with controls [21]. However, Giacobini [22] indicated that AChE levels decreased by as much as 90% compared with normal values in severe AD. Dekosky *et al.* [23] reported no change in ChAT activity in the inferior parietal, superior temporal, and anterior cingulate cortices in individuals with mild cognitive impairment (MCI) and mild AD compared with controls; ChAT activity in the superior frontal cortex was significantly elevated in subjects with MCI compared with normal controls, whereas no difference was observed between the mild AD group and the MCI and no cognitive impairment groups. Dekosky *et al.* [23] also found significantly higher hippocampal ChAT activity in subjects with MCI than in the control and AD groups. Thus, it can be speculated that significant reductions in ACh level and the activities of ChAT and AChE in mouse brains are likely related to differences in disease progression and among brain regions. Taken together, our results suggest that ChAT-positive cell loss in the MS and hippocampal CA1 regions of the AD brain occurs earlier than changes in the cholinergic system (ACh level and ChAT and AChE activities) in the cerebral cortex. In addition, different brain regions or progressions of AD may contribute to inconsistent alterations in ACh level and activities of ChAT and AChE.

SFN has many advantages, including water solubility, good pharmacokinetics, and safety after oral administration, as well as the potential ability to penetrate the blood-brain barrier [24,25]. Animal models of traumatic brain injury [26], idiopathic Parkinson’s disease [27], cortical neuron injury [28], and spinal cord injury [29] have suggested that SFN is an effective neuroprotector. Furthermore, we found that SFN might play a role in protecting against cognitive function impairment and cholinergic neuron loss in the brains of mice with AD-like lesions induced by combined administration of aluminum and d-galactose. The findings of a previous study and ours suggest that SFN is a promising compound with neuroprotective properties, which is expected to be useful in the prevention of AD [12,14].

Neurogenesis is maintained in two regions—the SVZ and SGZ—in the adult brain [30,31]. The proliferation of neurocytes in these regions has been studied in various AD mouse models, but the findings have been inconsistent. Although an overall trend of impaired neurocyte proliferation in these areas in AD has been documented [32,33,34,35], Kamphuis *et al*. [36] and Díaz-Moreno *et al.* [37] observed enhanced proliferation in APP/PS1 and SAMP8 mice, respectively. In our study, dramatic increases in the numbers of BrdU-immunoreactive cells in the SVZ and SGZ were observed in AD mice compared with controls. Differences in disease progression may contribute to the conflicting results obtained in various studies. Numerous studies have provided evidence that SFN can inhibit the proliferation of cancer cells [38,39,40]. In contrast, human mesenchymal stem cells incubated with low doses (0.25 and 1 μM) of *R*-SFN exhibited higher proliferation rates compared with controls, whereas 20 μM *R*-SFN induced a significant reduction in the proliferation index [41]. We observed more BrdU-immunoreactive cells in AD-like mice treated with 25 mg/kg d,l-SFN than in control and AD-like mice that received no treatment, suggesting that SFN can enhance the proliferation of neurocytes in the AD brain. This effect may partly account for the increased number of cholinergic neurons in AD-like mice with than in those without SFN treatment. As is well known, BrdU can be incorporated into DNA only during the *S*-phase of the mitotic process. Further research is thus needed to confirm these results using another marker, such as Ki-67, which is fully positive for its whole duration. Moreover, a Terminal-deoxynucleoitidyl Transferase Mediated Nick End Labeling (TUNEL) test is necessary to further validate the results by showing no change in the presence and absence of SFN in the mouse brain.

In the present study, a significant decrease in brain aluminum level was observed in AD-like mice treated with SFN. Moreover, SFN remarkably reduced the blood aluminum level in similar AD-like mice in our previous study. These findings suggest that SFN relieves the brain aluminum load by accelerating blood aluminum excretion, which could be partly ascribed to its ability to induce phase II detoxifying enzymes by activation of the transcription factor Nrf2 [42,43]. Therefore, aluminum load reduction may be implicated in SFN-mediated neuroprotective effects in AD-like mice. Furthermore, oxidative stress and inflammation play independent and/or dependent roles in the initiation and progression of AD [44,45]. The involvement of Nrf2 signaling in the regulation of oxidative stress and inflammation has been well documented [46,47]. As a potent activator of Nrf2, SFN may also play an important neuroprotective role against AD via anti-inflammation and/or antioxidation. It has been reported that SFN attenuates Aβ-induced oxidative cell death via activation of Nrf2 [11], and protects the brain from Aβ deposits and peroxidation in mice with AD-like lesions induced by combined administration of d-galactose and aluminum [14]. Therefore, further study is required to confirm whether the activation of Nrf2 signaling is involved in the anti-AD effect of SFN.

In conclusion, our study provides insight into the application of SFN to prevent and cure AD. However, the potentially neuroprotective mechanism of SFN against AD should be investigated further. Moreover, this study did not include control mice treated only with SFN, which limits the interpretation of the results.

## 3. Experimental Section

### 3.1. Reagents

d-(+)-Galactose (purity ≥ 98%) and aluminum chloride (purity ≥ 97.0%) were purchased from Sigma Chemical Corporation (St. Louis, MO, USA); d,l-sulforaphane (purity ≥ 97.0%) was purchased from Toronto Research Chemicals, Inc. (Toronto, ON, Canada); rabbit anti-ChAT polyclonal antibody (ab68779) was obtained from Abcam Inc. (Cambridge, MA, USA); and rabbit anti-BrdU polyclonal antibody (bs-0489R) was obtained from Bioss Biotechnology (Beijing, China). Immunohistochemistry kits were purchased from Beijing Zhongshan Biotechnology (Beijing, China). ACh, ChAT, and AChE test kits were obtained from Nanjing Jiancheng Institute of Biotechnology (Nanjing, China). All other reagents were of the highest purity available and were obtained from commercial sources.

### 3.2. Animals, Treatments, and Tissue Collection

Eight-week-old Kunming mice (animal code SCXK 2008-1105) were purchased from the Experimental Animal Center at China Medical University (Shenyang, China). The mice were maintained on a 12 h light/dark cycle with controlled temperature (22 ± 2 °C) and humidity (55% ± 15%), and provided with food and water *ad libitum*. The Animal Care and Use Committee of China Medical University approved the experiment (no. CMU20130105), which complied with the National Institute of Health’s Guide for the Care and Use of Laboratory Animals. All efforts were made to minimize suffering and the number of animals used.

**Figure 7:**
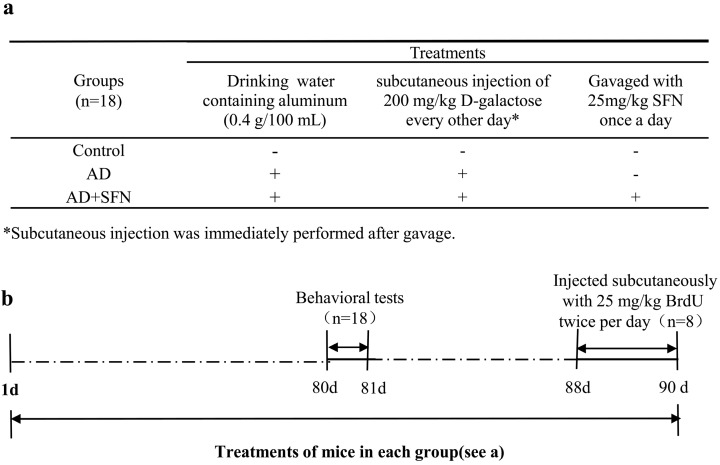
Scheme of animals groups and treatments. As shown in (**a**), mice were divided into 3 groups: control, AD-like and AD + SFN-like. Mice in groups of AD and AD + SFN were administered by the daily and free provision of drinking water containing aluminum (0.4 g/100 mL) and subcutaneous injection of 200 mg/kg d-galactose (dissolved in physiological saline) every other day. Distilled water and an equivalent amount of physiological saline were administered to mice in the control group as drinking water and by subcutaneous injection, respectively. Mice in the AD + SFN group were gavaged with 25 mg/kg sulforaphane (dissolved in distilled water) once a day, whereas mice in groups of AD and control were gavaged with an equivalent amount of distilled water; (**b**) shows that all mice were treated for 90 days. Behavioral tests were performed after 80 days of treatment. Three days before sacrifice, 25 mg/kg 5-Bromo-2'-deoxyuridine (BrdU) was subcutaneously injected twice per day to 8 mice in each group.

According to body weight, mice were randomly divided into 3 groups (*n* = 18, equal numbers of male and female mice): a control group and AD-like groups with and without SFN treatment. Animal groups and treatments are shown in Scheme (Figure 7). Mice in groups of AD-like with or without SFN treatment were administered by the daily and free provision of drinking water containing aluminum (0.4 g/100 mL) and subcutaneous injection of 200 mg/kg d-galactose (dissolved in physiological saline) every other day. Distilled water and an equivalent amount of physiological saline were administered to mice in the control group as drinking water and by subcutaneous injection, respectively. AD-like mice in the SFN treatment group were gavaged with 25 mg/kg SFN (dissolved in distilled water) once a day, whereas mice in the AD-like and control groups were gavaged with an equivalent amount of distilled water. All mice were treated for 90 days.

Behavioral tests were performed after 80 days of treatment. Three days before sacrifice, the thymidine analog BrdU (25 mg/kg, twice per day, injected subcutaneously) was co-administered to 8 mice in each group to measure ongoing cell proliferation. Twelve hours after final BrdU administration, killed the mice and collected the brains, which were used to investigate the numbers of ChAT-positive and BrdU-positive cells by immunohistochemical assays. The brains of the remaining 10 mice in each group were removed immediately and bisected. The left cerebra were weighed and aluminum level was measured by graphite furnace atomic absorption spectrometry. The right cerebral cortices were used to test ACh level and activities of ChAT and AChE.

### 3.3. Step-Down-Type Passive Avoidance Tests

Step-down-type passive avoidance tests were conducted using a YLS-3TB platform recorder (Yiyan Technology, Jinan, China) to investigate the effects of SFN on learning and memory according to a modification of the method reported by Ukai *et al.* [48] and Sakaguchi *et al.* [49]. The experimental device is a 12 × 12 × 18-cm electronic avoidance-response chamber, three sides of which are made of blank Plexiglas and one side of which is hard black plastic. The floor of the chamber is composed of parallel stainless-steel grids. Electric shocks were delivered to the grids. A rubber platform (5 cm high, upper surface 4 cm in diameter) was fixed in a corner on the floor of the chamber.

The test consisted of a training session and a retention session (24 h after the training session). During the training session, each mouse was placed on the steel grids and then exposed to an electric shock (30 V for 5 min) until it stepped up onto the rubber platform. Escape latency (the time required for the mouse to escape from electric shock) and the number of errors (the number of times that the mouse stepped down from platform) were recorded. In the retention test, each mouse was placed on the platform. When the mouse stepped down and placed its paws on the grids, an electric shock was delivered. Step-down latency and the number of errors were recorded. The cut-off time in both sessions was 300 s.

### 3.4. Aluminum Measurement by Atomic Absorption Spectrometry

The left cerebra of mouse brains were immediately dissolved in a solution (3 mL) consisting of concentrated nitric acid and 30% hydrogen peroxide. The solution was digested at 120 °C for 3 h, and diluted to 10 mL with aluminum-free water. Brain aluminum levels were determined by graphite furnace atomic absorption spectrometry (Hitachi, Tokyo, Japan) using a wavelength of 309.3 nm, slit width of 1.3 nm, lamp current of 10.0 mA, and injection volume of 10 μL.

### 3.5. Immunohistochemistry Assays

Mice were deeply anesthetized and transcardially perfused with saline followed by ice-cold 4% paraformaldehyde (pH 7.4). The brains were removed, post-fixed with the same fixative overnight at 4 °C, and embedded in paraffin. Coronal sections (5 μm) were dried well at 60 °C for 2 days, and then dewaxed in xylene and hydrated in an alcohol row, and washed 3 times in phosphate-buffered saline (PBS; pH 7.2).

Endogenous peroxidase activity was blocked by immersion in 3% hydrogen peroxide for 10 min. Sections were incubated in 0.3% Triton-100X and then washed in PBS. DNA denaturation for BrdU staining was carried out by incubating sections in 2 N HCl at 37 °C. All sections were then blocked in normal goat serum for 30 min at room temperature. Sections were incubated with rabbit anti-BrdU polyclonal antibody (1:200, bs-0489R; Bioss Biotechnology, Wuhan, China) or rabbit anti-ChAT polyclonal antibody (1:400, ab68779; Santa Cruz, TX, USA) overnight at 4 °C. After washing in PBS, sections were incubated with secondary antibody (goat anti-rabbit immunoglobulin G) for 30 min, then washed with PBS and incubated with horseradish peroxide avidin-biotin complex for another 15 min, followed by washing with PBS. The reaction products were visualized with diaminobenzidine chromogen solution. Sections were then counterstained with hematoxylin and numbers of ChAT-positive and BrdU-positive cells were counted under an optical microscope.

### 3.6. ACh Level and Activities of ChAT and AChE

ACh level and activities of ChAT and AChE in the cerebral cortex were measured by colorimetric diagnostic kits according to the manufacturer’s instructions. Total protein in supernatant was detected using the Coomassie brilliant blue method. ACh level was expressed as micrograms per milligram fresh tissue protein, and activities of AChE and ChAT were described in units per milligram fresh tissue protein.

### 3.7. Statistical Analyses

Data from step-down-type passive avoidance tests were expressed as medians and interquartile ranges, and statistical comparisons were made by Kruskal–Wallis non-parametric one-way analysis of variance (ANOVA), followed by the Mann–Whitney *U*-test (two-tailed). SPSS software (version 13.0; SPSS Inc., Chicago, IL, USA) was used for all analyses. Data from other indices were presented as means ± standard errors of the mean (S.E.M.) and compared using one-way ANOVA followed by *post hoc* least significant difference multiple comparison tests. Probability values <0.05 were considered to indicate statistical significance.

## 4. Conclusions

SFN ameliorates neurobehavioral deficits by reducing cholinergic neuron loss in the AD-like mouse brain, and the mechanism may be associated with neurogenesis and aluminum load reduction. These findings suggest that the phytochemical SFN has potential application in AD therapeutics.
